# Comparison of the Responses of Soil Fungal Community to Straw, Inorganic Fertilizer, and Compost in a Farmland in the Loess Plateau

**DOI:** 10.1128/spectrum.02230-21

**Published:** 2022-01-12

**Authors:** Yalin Yin, Ye Yuan, Xiaowen Zhang, Huhe, Yunxiang Cheng, Shinchilelt Borjigin

**Affiliations:** a School of Ecology and Environment, Inner Mongolia University, Hohhot, China; b Key Laboratory of Ecology and Resource Use of the Mongolian Plateau, Ministry of Education of China, Hohhot, China; c Collaborative Innovation Center for Grassland Ecological Security, Ministry of Education of China, Hohhot, China; d Biodiversity Division, National Institute for Environmental Studies, Ibaraki, Japan; Broad Institute

**Keywords:** loessial soil fungi, fertilization practices, community composition, co-occurrence pattern, potential function

## Abstract

The Loess Plateau is located in the arid and semi-arid regions in northern China. The ecosystem is particularly sensitive to natural and anthropogenic disturbances. Fungi can produce extracellular enzymes, decompose a variety of organic matter, and regulate carbon and nutrient balance. We studied the changes of soil fungal community compositions in response to straw, inorganic fertilizer, and compost in a typical farmland in the Loess Plateau. Our results demonstrated that the addition of straw significantly reduces the Shannon index of the fungal community, in addition, the participation of straw significantly affects the composition of the fungal community. Functional prediction based on FUNGuild showed that straw significantly reduced the relative abundance of saprotrophs, pathotrophs, symbiotrophs, lichenized, ectomycorrhizal, and plant pathogens. Although fertilization practices destroyed the co-occurrence pattern among the fungal species, the addition of straw alleviated this affect. No significant effect of straw, compost, and inorganic fertilizers on the co-occurrence pattern among species in the soil fungal community was observed. Compared with compost and inorganic fertilizer, the addition of straw shaped the community composition by changing the relative abundance of fungal functional taxa. Thus, in the fragile Loess Plateau environment, over-fertilizing or non-order-fertilizing may destroy the co-occurrence pattern of the fungal communities and Loess Plateau ecosystem.

**IMPORTANCE** Determining the response of soil fungi in sensitive ecosystems to external environmental disturbances is an important, yet little-known, topic in microbial ecology. In this study, we evaluated the impact of traditional fertilization management practices on the composition, co-occurrence pattern, and functional groups of fungal communities in loessial soil. Our results show that in the fragile Loess Plateau environment, fertilizer management changed the composition of the fungal community and disrupted the co-occurrence pattern between fungi. The application of straw alleviates the destroying of the co-occurrence pattern. The current research emphasizes the necessity of rational fertilization of farmland in loessial soil.

## INTRODUCTION

In soil ecosystems, soil microorganisms play an important role (1), participating in many important ecosystem processes, including the carbon cycle (2–3), nitrogen cycle (4–5), and so on. Microbial diversity is an important indicator of soil fertility and health (6); therefore, microorganisms are sensitive to environmental changes. Fertilizer management practices of agricultural land affects the growth and metabolism of soil microorganisms by changing the soil’s physical and chemical properties (7–8). This leads to a change in the microbial community composition and diversity, thus, affecting the biogeochemical cycles, energy flow, and ecological function of the whole farmland ecosystem (9–11).

Fungi are an important part of soil microorganisms and play a crucial role in soil ecosystems. Compared with bacteria, fungi can degrade complex compounds better (12). In addition, fungi are symbiotic with crops and form mycorrhiza. When the soil nutrients are poor, saprophytic fungi are the first to degrade the inert organic matter and release nutrients in the soil (13–14). The role of saprophytic fungi in low soil nutrient conditions is of great significance to plant growth and maintaining the stability of the agro-ecosystem (15). Fertilizers can have various effects on the soil fungal communities, for example, different types of fertilizer affect the soil nutrient status, which may directly or indirectly affect the soil fungal community (16). The application of nitrogen fertilizer reduces the fungal biomass (17), changes the composition, and reduces fungal diversity (18–19). Previous studies have shown that mineral fertilizers can inhibit mycorrhizal fungal growth (20–21), whereas organic fertilizers can promote mycorrhizal fungal growth (22–23). In general, fertilizers may have a significant impact on the composition and diversity of the soil fungal communities, especially in ecosystems that are highly sensitive to human activities (24).

The Loess Plateau is one of the most sensitive areas to natural and human disturbances (25). The main soil type is cultivated loessial soil, which is prone to water and soil erosion, and hence the ecological environment is fragile. However, the Loess Plateau has a long history of farming and a variety of fertilization practices, which also puts forward higher requirements for the rational use of fertilizer (26–27). Therefore, the evaluation of the response of soil microorganisms in the Loess Plateau to different fertilizers is essential for the sustainable development of farmlands in this region. Most previous studies have focused on the soil bacterial communities (28–30), while only few have focused on fungal communities, and there are few reports on the comparative analysis of various traditional fertilization management practices. Therefore, to simulate the local actual fertilization conditions, we selected wheat straw (WS), manure compost (MC), mineral fertilizer (CF), wheat straw plus mineral fertilizer (WSCF), and manure compost plus mineral fertilizer (MCCF) to conduct related research on a corn farmland, according to the traditional fertilization practice and soil properties of Loess Plateau.

This study aimed to (i) investigate the effects of traditional fertilization management practices on the composition and diversity of fungal communities; (ii) determine the principal controlling factors affecting the soil fungal community composition and to clarify the changes in the fungal community and its relationship with the soil's physical and chemical properties; and (iii) evaluate the response of fungal functional taxa and co-occurrence network to different fertilization management practices in the cultivated loessial soil. We hypothesize that fungi are sensitive to the input of organic straw matter because most fungi secrete extracellular enzymes that can decompose xylem fiber and other complex organic matter (31). To achieve these goals, a 3-year field experiment was conducted. This study will have implications for sustainable development of cultivated loessial soil farmlands.

## RESULT

### Soil characteristics.

The soil physicochemical properties of the different fertilization treatments are shown in Table 1. Applying inorganic fertilizer significantly affected the available phosphorus (AP) and available potassium (AK) contents. Specifically, compared with the unfertilized control (UC) treatment, the addition of mineral fertilizer (CF) (*P = 0.006*), wheat straw plus mineral fertilizer (WSCF) (*P = 0.014*), and manure compost plus mineral fertilizer (MCCF) (*P = 0.033*) on the AP content were significantly different. The effects of manure compost (MC) (*P = 0.003*) and MCCF (*P = 0.001*) on the AK content differed significantly from those of UC and other fertilization treatments. However, the effect of fertilization treatment on other physical and chemical indicators is not significant. The fertilization treatment improved soil fertility, but also led to a decreasing tendency for the soil water content.

**TABLE 1:** Soil physicochemical properties of different fertilization treatments^*a*^

Treatment	UC	WS	MC	CF	WSCF	MCCF
pH	8.52 ± 0.06	8.50 ± 0.02	8.45 ± 0.03	8.41 ± 0.08	8.49 ± 0.03	8.48 ± 0.03
Moisture (%)	8.96 ± 1.66	9.14 ± 0.81	7.14 ± 0.55	7.42 ± 0.49	6.31 ± 0.30	6.60 ± 0.58
TOC (g·kg^–1^)	0.39 ± 0.03	0.44 ± 0.06	0.44 ± 0.08	0.41 ± 0.07	0.44 ± 0.06	0.43 ± 0.02
TN (g·kg^–1^)	0.04 ± 0.00	0.06 ± 0.01	0.05 ± 0.00	0.04 ± 0.00	0.06 ± 0.01	0.05 ± 0.01
AP (mg·kg^–1^)	2.39 ± 0.98^a^	3.24 ± 1.95^ab^	7.93 ± 4.83^ab^	10.87 ± 3.51^b^	8.86 ± 2.26^b^	12.98 ± 2.51^b^
AK (mg·kg^–1^)	155.77 ± 16.84^a^	153.93 ± 30.62^a^	228.42 ± 11.67^b^	175.62 ± 2.70^a^	167.24 ± 17.55^a^	241.62 ± 22.52^b^
NO_3_^–^ (mg·kg^–1^)	4.76 ± 1.11	5.89 ± 1.81	3.96 ± 0.41	7.92 ± 2.18	8.10 ± 3.59	11.26 ± 4.59
NH_4_^+^ (mg·kg^–1^)	1.84 ± 1.09	2.10 ± 1.03	5.19 ± 2.18	1.91 ± 0.19	2.13 ± 0.00	2.94 ± 0.34
C:N (%)	8.74 ± 0.40	7.97 ± 0.45	8.41 ± 1.26	9.18 ± 1.21	7.65 ± 1.20	7.93 ± 0.67

a UC, unfertilized control; WS, wheat straw; MC, manure compost; CF, mineral fertilizer (nitrogen, phosphorus, and potassium); WSCF, WS plus CF; MCCF, MC plus CF; TOC, total organic carbon; TN, total nitrogen; AP, available phosphorus; AK, available potassium; NO_3_^–^, nitrate nitrogen; NH_4_^+^, ammonium nitrogen. The physicochemical properties in the table are average value ± standard errors, the same letter indicates no significant difference, and different letters indicate a significant difference (*p* < 0.05), the others are not marked with letters are not significant.

### Soil fungal community composition.

**FIG 1:**
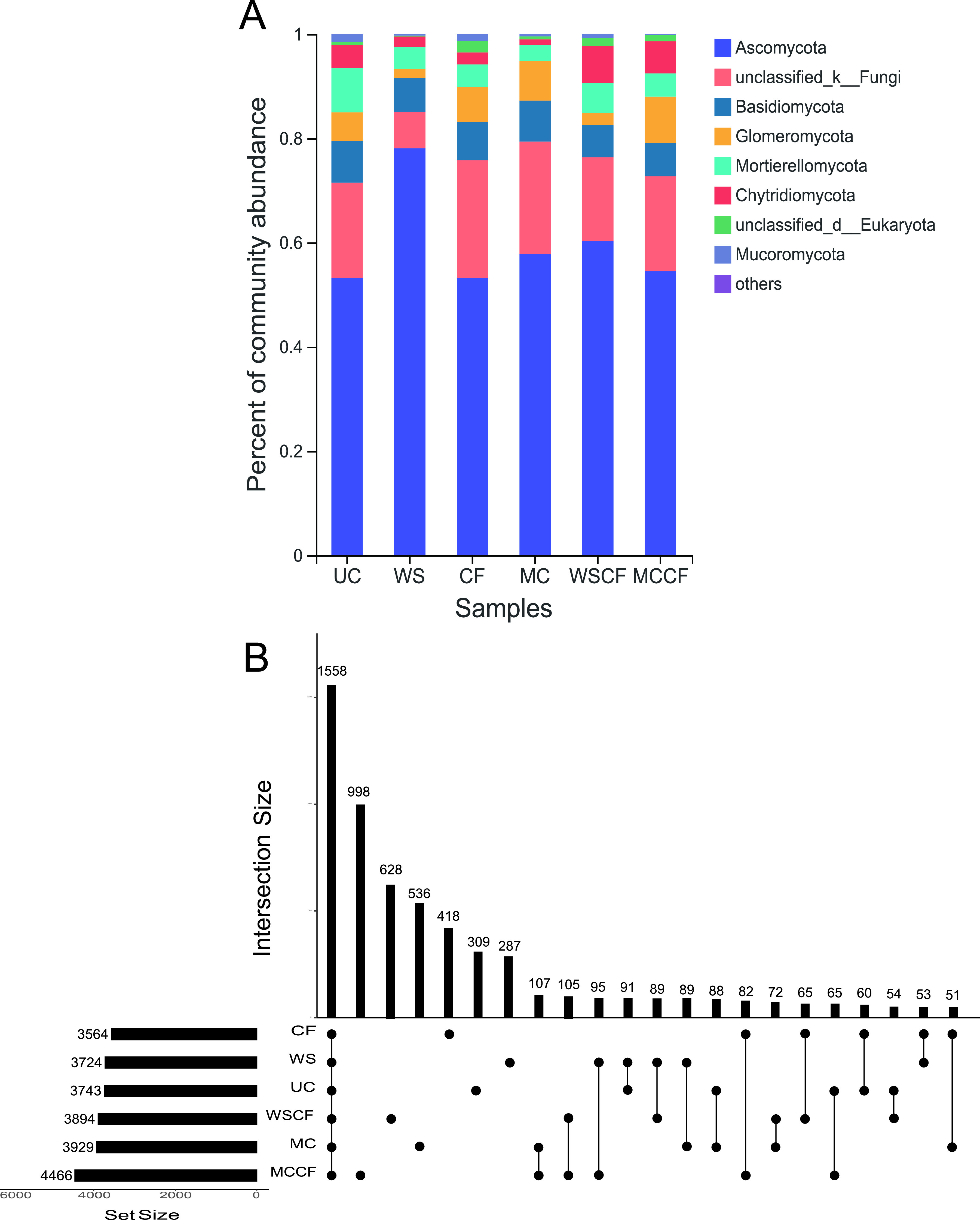
Variation in compositions of fungal communities: (A) the relative abundance of soil fungal phylum under different fertilization treatments; and (B) the Upset-Venn diagram of community species composition under different fertilization treatments.

Ascomycota is the dominant fungus in loessial soil, and its relative abundance ranged between 53.13% and 78.09% in the six fertilization treatments. The remaining relative abundances greater than 1% are mainly Basidiomycota, Glomeromycota, Mortierellomycota, and Chytridiomycota. Different fertilization treatments have different degrees of influence on the fungus phylum (Fig. 1A). Specifically, compared with the UC treatment, Ascomycota increased by 47.16% after the WS treatment. Interestingly, Glomeromycota decreased by 67.81% and 57.37% after the WS and WSCF treatment, respectively; Chytridiomycota was affected by the single fertilizer treatment, the WS, MC, and CF treatments reduced it by 54.57%, 74.66%, and 47.49%, respectively.

The results of the Upset-Venn analysis of community species composition of the samples under different fertilization treatments are shown in Fig. 1B. The results showed that the operational taxonomic unit (OTU) of the MCCF treatment is the highest. Except for the 1,558 OTUs shared, there were more unique species in the fungal communities under different fertilization treatments, indicating that different fertilizers enriched the corresponding fungal communities. The number of shared OTUs among the treatments involving organic fertilizers (such as MC and MCCF, WSCF and MCCF, WS and MCCF, WS and WSCF, WS and MC) was higher than the number of shared OTUs among other treatments.

### Analysis of soil fungal community diversity under different fertilization treatments.

**FIG 2:**
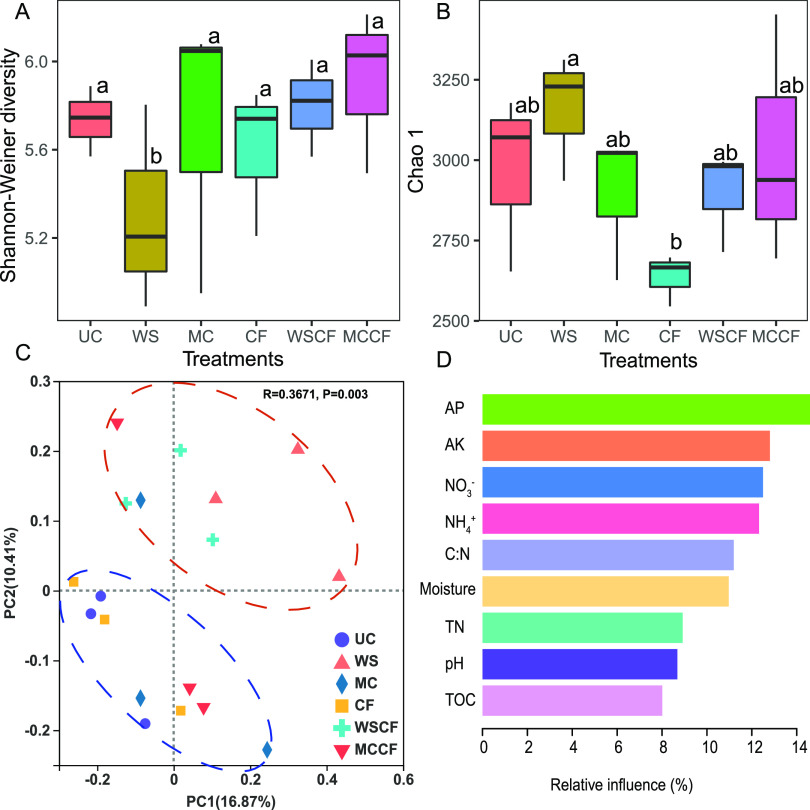
Effects of different fertilization treatments on the α and β diversity indices of soil fungi: (A) analysis of variance (ANOVA) of the Shannon Index; and (B) ANOVA of the Chao1 index. The same letter means no significant difference, and different letters mean significant difference (*p* < 0.05); (C) principal coordinates analysis (PCoA) of soil fungal communities under different fertilization treatments. The values of axes 1 and 2 are the percentages that can be explained by the corresponding axis; and (D) aggregated boosted tree (ABT) analysis showed the relative influence of changes in soil physical and chemical factors on the fungal communities in loessial soil after fertilization. TOC, total organic carbon; TN, total nitrogen; AP, available phosphorus; AK, available potassium; NO_3_^–^, nitrate nitrogen; NH_4_^+^, ammonium nitrogen.

The Shannon and Chao1 indices (α diversity) of the fungal communities under different fertilization treatments are shown in Fig. 2A and B. In addition to WS treatment, the response of the Shannon index of the fungal community to other fertilization treatments was not evident. There is a significant difference in fungal community richness index (Chao1) between WS and CF treatments. Thus, it was speculated that their effect on the Chao1 index of the fungal community in the cultivated loessial soil was the opposite. The average sequencing depth is about 60,000 reads. Dilution curve analysis showed that the sequencing of all investigated communities was close to saturation at 3% genetic distance, and the sequence number could reflect the diversity of the soil fungal communities (Fig. S1). The number of OTUs observed in the experiment was consistent with the diversity data predicted by the Shannon index.

The principal co-ordinates analysis-β diversity (PCoA) showed that the six treatments were divided into two groups (Fig. 2C): that is UC, MC, CF, and MCCF groups, and WS and WSCF as the other group, suggesting that straw had a significant impact on the composition of the soil fungal community. Furthermore, the permutational multivariate analysis of variance (PERMANOVA) test also yielded the same results (Table 2). Aggregated boosted tree (ABT) model was employed to interpret the relative importance of soil characteristics to the composition of the soil fungal community after fertilization (Fig. 2D). Soil properties, such as AP and AK, are the most accurate predictors of change in fungal community composition, with a cumulative relative contribution rate of 26%.

**TABLE 2:** The permutational multivariate analysis of variance (PERMANOVA) analysis to several kinds of fertilization treatments^*a*^

Characteristics	R^2^	*P*-value	*P*-adjust
CF	0.07355	0.105	0.138
WS	0.11045	***0.002***	***0.004***
MC	0.07133	0.138	0.138

a The significant influences of different fertilization on fungal communities are indicated in bold italics. WS, wheat straw; CF, mineral fertilizer; MC, manure compost.

### Classified biomarkers of soil fungal community.

**FIG 3:**
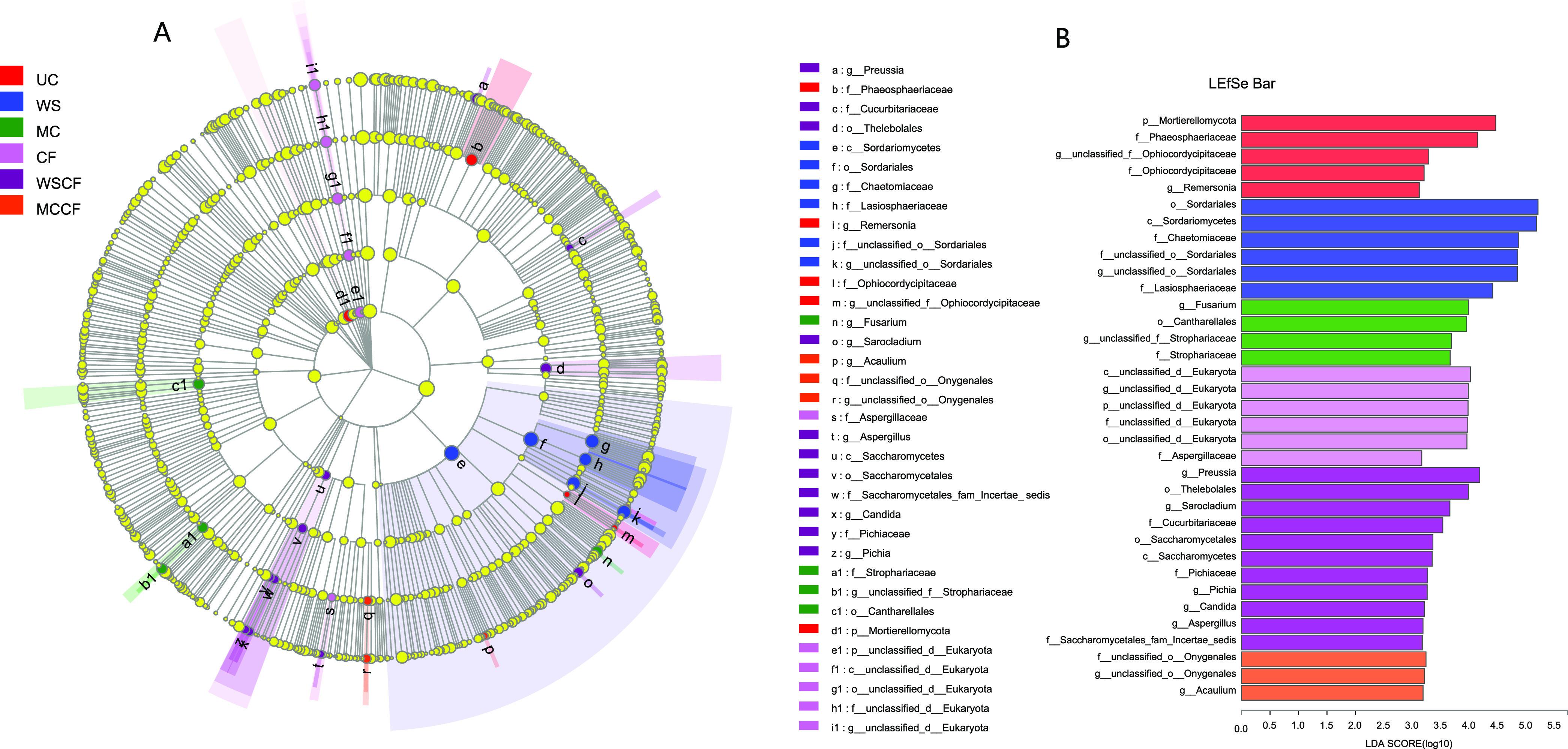
LEfSe species variances analysis of soil fungal in different fertilization treatments: (A) multi-level species hierarchical tree. Areas of different colors represent different methods of fertilization. Circles represent the level at which the system develops from domain to genus, and the diameter of each circle is proportional to the abundance of the group; (B) fungal groups with significant differences in abundance between communities. Horizontal coordinates represent linear discriminant analysis (LDA) values, and vertical coordinate represent fungal species with significant group differences (LDA score ≥ 3). Yellow nodes indicate no significant differences between groups (*P* > 0.05), and red, blue, green, light purple, purple, and orange nodes represent different species with higher abundance in the UC, WS, MC, CF, WS plus CF, and MC plus CF, respectively.

Besides determining the α and β diversity, another major goal of comparing microbial communities is to identify specific communities in the samples (32). Therefore, through LEfSe analysis (LDA threshold is 3), we founded 35 biomarkers belonging to four phyla are sensitive to different fertilizer treatments in soils (*P < *0.05; Fig. 3A), Mortierellomycota, Phaeosphaeriaceae, unclassified Ophiocordycipitaceae (from family to genus), and Remersonia (with undetermined status in the Sordariomycete) which was identified as the most abundant biomarker in UC soil. Sordariomycetes (from class to genus), Chaetomiaceae, and Lasiosphaeriaceae are particularly abundant in WS. Fusarium, Cantharellales, and Strophariaceae (from family to genus) are significantly enriched in MC soil; unclassified Eukaryota (from phylum to genus) and Aspergillaceae are more sensitive to mineral fertilization. Preussia, Thelebolales, Sarocladium, Cucurbitariaceae, Saccharomycetes (from class to genus), and Aspergillus are significantly enriched in WSCF soil. Onygenales (from order to family) and Acaulium are more sensitive to MCCF fertilization. These were the main groups leading to the differences in the cultivated loessial soil fungal communities under the different fertilization treatments (Fig. 3B).

### Co-occurrence pattern of soil fungi under different fertilization treatments.

**FIG 4:**
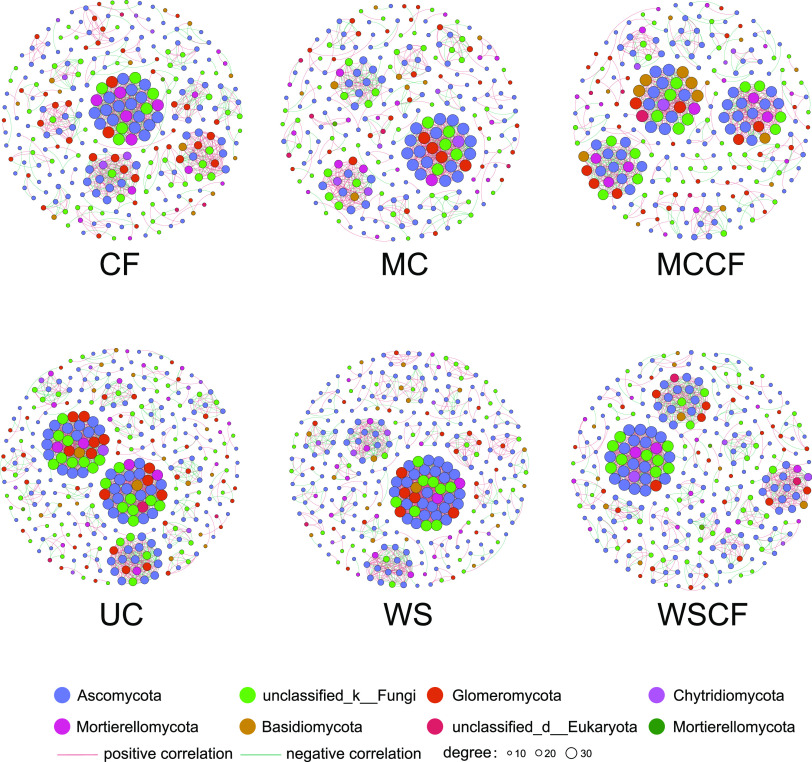
Co-occurrence network analysis of cultivated loessial soil fungi community under different fertilization. The color of each node represents a phylum of fungal species; each node represents an operational taxonomic unit (OTU); the node size is proportional to the abundance of the classification unit; the red connection represents a positive correlation; and the green connection represents a negative correlation, circles of different sizes represent the degree of nodes.

To determine the effect of fertilization on the co-occurrence pattern among species in the soil fungal community, we constructed fungal community co-occurrence network under several fertilization treatments (WS, MC, CF, WSCF, and MCCF). Compared with the UC group, the nodes, edges, and mean degree of the network treated with WS, MC, CF, WSCF, and MCCF decreased. Such as the mean degree, WS, MC, CF, WSCF, and MCCF group was decreased by 0.29, 3.07, 2.29, 1.81, and 2.09, respectively, the clustering coefficients are reduced by 0.02, 0.05, 0.05,0.01, and 0.06, respectively. This indicates fertilization management destroyed the co-occurrence pattern of soil fungi. Interestingly, the ratio of positive and negative correlations increased from 1.14 to 2.21 after fertilization. Although the co-occurrence pattern between the fungi is destroyed, the symbiosis is enhanced. However, after adding WS, whether inorganic fertilizer existed, the destroying of co-occurrence pattern was alleviated (Fig. 4, Table 3).

**TABLE 3:** Topology index of each network in Fig. 4^*a*^

Metric	Definition	Ecological relevance	UC	WS	MC	CF	WSCF	MCCF
Nodes	Each node represents a fungal operational taxonomic unit (OTU).	Larger networks contain a greater no. of interacting (co-occurring or co-excluding) OTUs.	333	323	253	276	296	244
Edges*^b^*	Edges indicate significant cooccurrence or co-exclusion relationships.	Co-occurrence could represent a no. of ecological interactions, from predator-prey relationships to commensalism to shared ecological niches. Co-exclusion may represent competition or inhibition.	1,590 848 (53.21%) 742 (46.79%)	1,495 924 (61.81%) 571 (38.19%)	820 556 (67.80%) 264 (32.20%)	1,002 578 (57.68%) 424 (42.32%)	1,146 654 (57.07%) 492 (42.93%)	910 554 (60.88%) 356 (39.12%)
Mean degree	Degree refers to the no. of edges a given node has. Mean degree is the average degree across all nodes in a network.	Higher mean degree indicates more co-occurrence or co-exclusion relationships per OTU.	9.55	9.26	6.48	7.26	7.74	7.46
Density	Density is defined as the ratio of the no. of edges in a given network to the no. of edges possible for that many nodes.	High-density networks contain a large proportion of interacting OTUs.	0.025	0.023	0.02	0.023	0.024	0.024
Clustering coefficient	How nodes are embedded in their neighborhood, and thus the degree to which they tend to cluster together.	High clustering coefficient more tightened microbiome associations.	0.69	0.67	0.64	0.64	0.68	0.63

a UC, unfertilized control; WS, wheat straw; MC, manure compost; CF, mineral fertilizer (nitrogen phosphorus and potassium); WSCF, WS plus CF; MCCF, MC plus CF.

b The first and second lines are positive and negative edge proportions, respectively.

### Potential functional taxa of soil fungal community.

**FIG 5:**
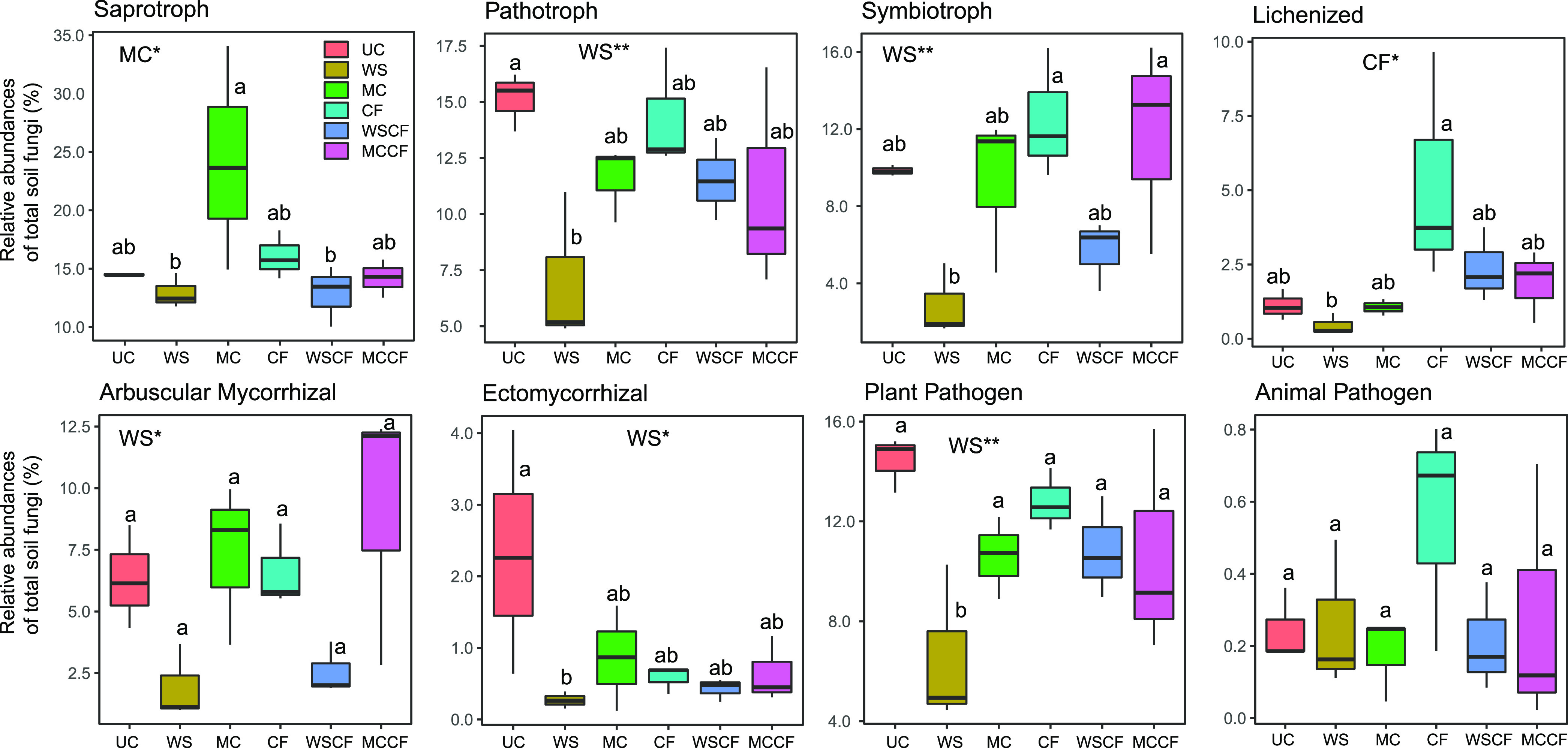
Ecological functional taxa of soil fungi under different fertilization treatments. UC, unfertilized control; WS, wheat straw; MC, manure compost; CF, mineral fertilizer (nitrogen phosphorus and potassium); WSCF, WS plus CF; MCCF, MC plus CF. Fertilization practices that have a significant effect on fungal functional taxa are marked on the top of the corresponding graph (*, 0.01 < *P ≤ *0.05; **, 0.001 < *P ≤ *0.01).

Compared with the UC group, the relative abundance of most hypothetical fungal functional taxa was not affected by the MC, CF, WSCF, and MCCF treatments (Fig. 5). However, WS significantly reduced the relative abundance of pathogen (*F* = 11.564; *P = *0.005), symbiotroph (*F* = 6.671; *P = *0.025), arbuscular mycorrhiza (*F* = 3.408; *P = *0.05), ectomycorrhiza (*F* = 10.103; *P = *0.008), and plant pathogens (*F* = 13.781; *P = *0.003) in the soil fungal community (Fig. 5). The reduction rates of these fungal potential functional taxa in the soil were 53.63%, 69.37%, 88.38%, 70.86%, and 54.55%, respectively. In addition, MC and CF significantly increased the relative abundance of saprotrophs (*F* = 7.88; *P = *0.016) and lichenization (*F* = 8.049; *P = *0.015), 1.67 and 4.67 times the UC, respectively. In this study, the fertilization category with the most significant effect on the fungal functional taxa of cultivated loess soil was WS. Whereas, WS had no significant effect on corn yield, other fertilization significantly increased corn yield (Fig. S3). The yield increase rates of MC, CF, WSCF, and MCCF are 36.41%, 87.23%, 74.65%, and 63.20%, respectively.

## DISCUSSION

Agricultural and animal husbandry production activities often affect the ecosystem to varying degrees, and fertilization management practices are one of the main factors affecting the stability and sustainability of agro-ecosystems (33). Fertilization and types of fertilization are closely related to the number, composition, and diversity of soil fungi (34–35). Our results showed that straw had the opposite effect on the Shannon and Chao1 indices of the fungal community (Fig. 2A and B). The decrease in fungal diversity caused by fertilization may be attributed to the “preference” effect (36). The rapid growth of the fungal groups causes an increase in nutrient consumption, which inhibits the growth of other fungal groups and leads to the decrease in fungal community diversity. Previous studies (37–38) have also shown that different fertilization patterns significantly changed the composition and abundance of fungal communities. The effect of different fertilization on the AMF (arbuscular mycorrhizal fungi) community of maize rhizosphere soil on the typical black soil in Northeast China showed that different types of fertilization (containing urea [N]; superphosphate [P; potassium sulfate [K], and organic manure) systems had a significant impact on the maize rhizosphere AMF diversity (39). However, the results of studies on the effects of fertilization on fungal diversity were different. Francioli et al. (40) found that the combined application of organic and inorganic fertilizers had no significant effect on the soil fungal community diversity in a farmland in central Germany. The effects of fertilization on the taxonomic composition and diversity of fungus community were inconsistent, which may be related to the type of fertilization, wherein inorganic fertilizers can easily lead to soil acidification and inhibit the growth of fungal communities (41). Whereas organic fertilizers do not lead to soil acidification (42). Our study found that in the cultivated soil of the Loess Plateau, the response of fungal communities to straw was very strong (Fig. 2C), which was related to the components of straw, such as cellulose, hemicellulose, and lignin (43). Compared with other microbial groups, fungi can use extracellular enzymes to degrade high-molecular organic substances more effectively (44) and therefore have a strong ability to decompose lignocellulose (45–46). Corn straw returning to the field can promote the abundance of fungi in Shajiang black soil and improve the soil structure (47), returning straw to the field can also increase the diversity of soil fungi, in which the fungal abundance and Shannon diversity index both increase with the increase in the amount of corn stalks returned. In addition, changes of the soil fungal community composition after fertilization were largely associated with by AP and AK (Fig. 2D). Our results are mutually supportive with the analysis of soil physicochemical properties (Table 1). Research on the effects of soil properties on fungal community composition has also found that AK and AP have important effects of soil properties on fungal community composition (48). However, a positive relationship between fungal growth and N-concentration (49) or inorganic N-addition in soil has also been previously reported (50). Thus, soil environmental factors can effectively explain the differences in microbial communities, and soil nutrients are significantly affected by fertilization, thereby affecting the characteristics of soil fungal communities. Regarding the effect of fertilization on corn yield, MC, CF, WSCF, and MCCF significantly increase the yield of corn, because CF and MC contain nutrients necessary for plant growth. At the same time, the combined application of fertilizers can provide balanced nutrients for crop growth (51).

Fertilization practices significantly affected the fungal groups with different ecological functions. Compared with composting and inorganic fertilizer, WS had the most significant effect on the fungal functional taxa. The significant decrease in the relative abundance of pathotrophic and symbiotic nutritional fungi may be due to the straw that weakens the ability of soil fungi to mineralize organic matter (52). Applying compost and inorganic fertilizer provides sufficient nutrients (53) for the growth of saprotroph fungi communities and promotes the growth and reproduction of these groups, which increases most in the MC group. Moreover, in the MCCF treatment, the abundance of arbuscular mycorrhizal fungi increased, and there was a common symbiotic relationship between the arbuscular mycorrhizal fungi and plants (54), the key role of arbuscular mycorrhizal fungi in promoting plant nutrient uptake has been confirmed in many studies (55–56). Soil C:N and moisture are considered to be the key limiting factors for the growth of arbuscular mycorrhizal fungi, and the change in available carbon, nitrogen, and phosphorus reservoirs under fertilization plays an important role in fungal growth, especially in semi-arid areas, and high C:N or high nitrogen content promotes its growth (57). Thus, arbuscular mycorrhizal fungi has a higher relative abundance in the MC and MCCF treatments. Spearman rank correlation analysis showed that Basidiomycota was significantly positively associated with C:N and NH_4_^+^ (Fig. S2A). Based on the analysis of the similarities and differences of soil fungi under different fertilization practices, the cultivated loessial soil fungal communities are dominated by Ascomycota and Basidiomycota. Studies in wheat-corn farmland in Poland have also shown that the dominant flora in the soil fungi is Basidiomycetes (58). Most Ascomycetes and Basidiomycetes employ saprophytic or symbiotic nutritional approaches (38) and play important roles as decomposers in the soil. Except for the WS treatment, other fertilization treatments increase the content of saprotroph fungi. The changes in the fungal community composition and potential function were consistent after fertilization. Adding inorganic fertilizer (CF, WSCF, and MCCF) increased the relative abundance of lichenized symbiotic fungi, which plays an important role in crop health and nutrition (59). Spearman rank correlation analysis also showed that lichenized symbiotic fungi were significantly positively correlated with the AP content (Fig. S2B). After fertilization, the relative abundance of plant pathogens decreased, while the effect of WS treatment was the most evident, and its change in the relative abundance affected the amount of pathotrophs; pathotroph and plant pathogens had the same change trend (60). Therefore, straw and the combined application of organic and inorganic fertilizers is a more beneficial fertilization method for soil and plants, which is beneficial for maintaining the soil health and diversity of the fungal community in cultivated loessial soil farmland (61).

The co-occurrence network analysis of the soil fungal community explored the effect of fertilization practices on the symbiotic model of the fungal community. Fertilization treatment reduced the connectivity of nodes in the fungal network, and fungal co-occurrence patterns were destroyed under the fertilization treatments (Fig. 4), especially in the MCCF treatment. The positive and negative correlations among species in the network can indicate the cooperative and competitive predation relationship among taxa. The increase in the proportion of negative links may indicate an increase in the negative interaction in the community (62), and this negative interaction between soil microorganisms may be caused by competition (63). A typical example of competition between species is the niche theory based on the “Gauss hypothesis,” that is, the same niche cannot coexist (64). The significant correlation ratio of fungal community after each fertilization treatment (Table 3) shows that the competitive relationship of the fungal community in soil is weakened after fertilization. Other studies have reached a similar conclusion that fertilization (whether added with mineral fertilizers or manure) reduced the complexity of the fungal network (65–66). Yao et al. (67) found that adding high amounts of nitrogen fertilizer simplified the ecological network structure of microorganisms and that is, the co-occurrence pattern was destroyed. Fertilization destroyed the co-occurrence pattern of within soil microbial community, which is consistent with our results. However, some studies have found that the increase in nutrients can improve the co-occurrence network complexity of the fungal community, such as adding phosphorus (68) and the mixed implementation of nitrogen fertilizer and straw (69). These studies found that with the increase in fertilizer application, the direct effect among fungal species increases, whereas the indirect effect decreases, and the increase in co-occurrence pattern would lead to a more stable network and stronger “resistance” to external interference. Therefore, we speculate that fertilization increases the nutrient supply and reduces the difficulty for organisms to obtain nutrients. The organisms need not consume energy to maintain close contact with other organisms to obtain nutrients; hence, the community can be more independent and effectively use the existing substrates in the habitat to survive (70). After fertilization, fungi may redistribute their resources by altering the nutrient cycles and the decomposition of organic matter, thus changing the network relationships (71). All types of organic matter are different under different soil conditions, and the co-occurrence pattern between fungi is closely related to the content of soil nutrients, soil matrix will lead to differences in fungal communities and have different effects on their co-occurrence pattern (72).

### Conclusions.

This study indicated that the fungi in cultivated loessial soil were mainly composed of Ascomycetes and Basidiomycetes, and fertilization practices significantly changed the fungal community composition. Functional taxa were more sensitive to fertilization than community composition, and different ecological functions had different responses to different category of fertilizer: WS fertilization significantly decreased the relative abundance of pathotroph fungi, whereas MC and CF significantly increased the relative abundance of saprophytic and lichenized symbiotic fungi, respectively. The WS treatment had the most significant effect on fungal function. In addition, AP and AK have been identified as the most accurate predictors of change in fungal community composition. The fungal co-occurrence pattern was destroyed under the fertilization treatments; however, after adding straw, whether inorganic fertilizer existed, the destroying of the co-occurrence pattern was alleviated. Therefore, a reasonable fertilization model is very important for the sustainable development of cultivated loessial soil, the specific effects of fertilization on soil carbon decomposition and nitrogen cycle need to be further studied.

## MATERIALS AND METHODS

### Field site and experiment design.

The experiment was carried out in the Dingxi Experimental Station of the Gansu Academy of Agricultural Sciences (Dingxi City, Gansu Province; 35° 35' N, 104° 36' E) in 2013 to 2015, which was characterized by a temporarily semi-arid climate, an altitude of 1,970 m, an average annual temperature of 6.2°C, an annual number of hours of sunup to 2,500 h, and a frost-free period of 140 d. The crops in the experimental site were harvested once a year, and there was no irrigation, which was classified as a typical rain-fed agriculture in dry land. The average annual precipitation was 415 mm. Precipitation from June to September accounted for 68% of the annual precipitation, and the relative variability of precipitation was 24%. The soil in the experimental area is cultivated loessial soil, the average bulk density of the 0 to 30 cm soil layer was 1.25 g/cm^3^, the field water holding capacity was 21.2%, and the permanent wilting coefficient was 7.2%.

Six plots with the same soil type and continuous geographical location were assigned to set up different fertilization practices, each treatment randomly established three replicates. The area of each sample plot was 6 m × 5 m to cultivate corn. The six management regimes were UC, WS (approximately 2 cm in length), MC, CF (N, P, K), WS plus CF (WSCF), and MC plus CF (MCCF). The amount of fertilizer applied as part of the different treatments is shown in Table S1, the nitrogen content of WS was 6.1 g kg^−1^ and the nitrogen content of MC is 22 g kg^−1^, further fertilizing was conducted in accordance with Table S1 before sowing every year.

### Soil sampling and analysis.

Soil samples were collected during the corn scion period in August 2015, using a 5-point sampling method. At each sampling plot, five surface soil samples (0 to 10 cm) were taken and mixed as one soil sample. The coarse impurities such as the root system and stone were removed using a 4-mm soil sieve and sealed in a plastic bag with zipper. Part of the soil samples were immediately placed in a liquid nitrogen tank and then stored in a low-temperature refrigerator in the laboratory (–80°C) for DNA extraction; another part of the soil samples was stored in a refrigerator at –20°C for extraction and determination of soil moisture and nitrate and ammonium nitrogen contents; the rest of the samples were dried naturally, and the physical and chemical characteristics of the soil were determined after grinding.

### Determination of soil physical and chemical properties.

A Starter-2100 pH probe was used to measure the soil pH (Ohaus, Brooklyn, NY, USA) in a soil-to-water ratio of 1:2.5 (wt/vol) soil solution (0.01 CaCl_2_). After drying at 105°C for 24 h, the soil moisture content was calculated. Soil total nitrogen (TN) was determined using Kjeldahl digestion, distillation, and titration (73). Water and inorganic carbon were removed by reacting the sample with phosphoric acid (74), and the soil organic carbon (TOC) was determined using the dry combustion method using an NC analyzer (Sumigraph NC-900; Sumika Chemical Analysis Service, Tokyo, Japan). After perchloric acid digestion and ascorbic acid reduction, AP was determined using molybdic acid colorimetry (75), and AK was analyzed using a flame atomic absorption spectrophotometer (76). The soil was extracted with KCl, the contents of soil nitrate nitrogen (NO_3_^–^-N) and ammonium nitrogen (NH_4_^+^-N) were determined, and an automatic flow analyzer (FIAstar 5000 analyzer; Foss Tecator, Hillerød, Denmark) was used for analysis (77).

### DNA extraction and high-throughput sequencing.

Following the manufacturer’s instructions, using the PowerSoil DNA isolation kit (Mo Bio Laboratories, Solana Beach, CA, USA), 0.5 g of soil was weighed to extract the total DNA. To detect the extraction quality of DNA in the sample, 1% agarose gel electrophoresis was used, and the concentration and purity of DNA were determined using a NanoDrop 2000 UV–vis spectrophotometer (Thermo Scientific, Wilmington, USA). To amplify the appropriate size of fungal fragments for the HiSeq analysis, PCR analyses were performed using a GeneAmp 9700 PCR thermocycler (Applied Biosystems, Foster City, CA, USA), using ITS1F (5′-CTTGGGCATTTAGAAGGAAGTAAMAL-3′); and ITS2R (5′-GCTGCGTTCTTCATCGATG-3′) (78), the DNA of the ITS1 region was amplified using PCR; this was repeated three times to prevent bias in PCRs. The PCR mixtures contained 0.25 μL of HotStarTaq polymerase (Qiagen, Valencia, CA, USA), 2.5 μL of 10× PCR buffer supplied by the manufacturer, 2.5 μL 10× deoxyribonucleotides triphosphates (dNTPs; 200 mM) each, 0.2 μL of 50 μM reverse primer, 1 μL of 10 μM forward primer, 0.25 μL of 100 mg ml^−1^ bovine serum albumin (BSA), 5 μL DNA template in a final volume of 25 μL. The PCR amplification of ITS1 rRNA gene was performed as follows: initial denaturation at 95°C for 15 min, followed by 35 cycles of denaturing at 94°C for 60 s, annealing at 51°C for 60 s and extension at 72°C for 60 s, and a final extension at 72°C for 10 min. PCRs were performed in triplicate. The PCR product was extracted from 2% agarose gel and purified using the AxyPrep DNA Gel Extraction Kit (Axygen Biosciences, Union City, CA, USA) according to manufacturer’s instructions and quantified using Quantus™ Fluorometer (Promega, USA). The DNA of the samples was sequenced on an Illumina HiSeq 2500 platform (Illumina, San Diego, USA) according to the standard protocols by Novogene (Beijing, China).

First, the raw ITS1 rRNA gene sequencing reads were quality-filtered using fastp version 0.20.0 (79), sequences shorter than 200 bp, ambiguous bases, and sequences with an average mass less than 25 were removed. After quality filtering, a fungal DNA sequence with an average length of 230 bp was obtained. The sequencing reads of each sample ranged from 51,521 to 97,648, and the OTU from all 18 samples ranged from 3,564 to 4,466, with a 97% sequence similarity cut-off. The remaining high-quality sequences were clustered into different operational taxa at a similarity level of 97% using USEARCH (version 7.1) (80); the compared database is UNITE ITS database (http://unite.ut.ee/index.php). Then we use the RDP classifier Bayesian algorithm to perform species taxonomic analysis on the representative sequence of OTU and count the community composition of each sample at different species classification levels.

### Statistical analysis.

One-way analysis of variance (ANOVA) of soil physical and chemical properties was performed using SPSS (version 26.0; SPSS, Chicago, IL, USA). Significance was calculated using Tukey’s test (*P < *0.05). The α diversity index (Shannon, Chao1) was calculated using mothur (version 1.30.1) (81). PCoA was performed at the OTU level, based on the Bray-Curtis distances. The Permutational multivariate analysis of variance (PERMANOVA) was used to compare the effects of different fertilization treatments on fungal communities and performed with 999 permutations using the Adonis function. The straw application variables were created by assigning the value 1 to the WS and WSCF treatments, and 0 to other treatments, compost application and mineral fertilizer application are the same as the analysis of straw application. ABT was used to quantitatively evaluate the relative influence of changes in soil physical and chemical factors on the fungal community in loessial soil after fertilization. OTU distribution Upset-Venn diagram, fungal community composition histogram, PCoA, PERMANOVA and ABT analysis were conducted using the “vegan” package of R software, version 3.5.1. Linear discriminant analysis (LDA) coupled with effect size measurements (LEfSe) analysis was conducted to search for statistically different biomarkers between groups (82). The network analysis was performed using open network analysis pipeline (Molecular Ecological Network Analyses Pipeline, http://ieg2.ou.edu/MENA/main.cgi) to better comprehend the interrelationship and co-occurrence pattern within the fungal community. We used an ensemble approach based on the four measurements, including Pearson and Spearman correlations and Bray-Curtis and Kullback-Leibler dissimilarities between pairwise OTUs (83). We filtered the correlation data with a cut-off at an absolute r-value of 0.7 and then used a significant *P* value of < 0.01 to filter the data from the previous step to improve the reliability of the networks. The potential ecological function categories of fungi in the cultivated loessial soil under different fertilization treatments were predicted by comparing the data of fungal OTUs with the FUNGuild (https://github.com/UMNFuN/FUNGuild) database (84). Using SPSS interactive analysis, the significant effects of different fertilization treatments on functional taxa were obtained.

### Accession number(s).

The raw reads were deposited into the NCBI Sequence Read Archive (SRA) database (Accession Number: SRP318718).
