# Supplementary material for: Comparison of the Responses of Soil Fungal Community to Straw, Inorganic Fertilizer, and Compost in a Farmland in the Loess Plateau
Source: Microbiol Spectr. 2022 Jan 12;10(1):e02230-21. doi: 10.1128/spectrum.02230-21 (PMC8754151; doi:10.1128/spectrum.02230-21)
Supplement: SUPPLEMENTAL FILE 1 — Supplemental material. Download SPECTRUM02230-21_Supp_1_seq4.pdf, PDF file, 0.3 MB [file spectrum02230-21_supp_1_seq4.pdf]

Supplementary Material

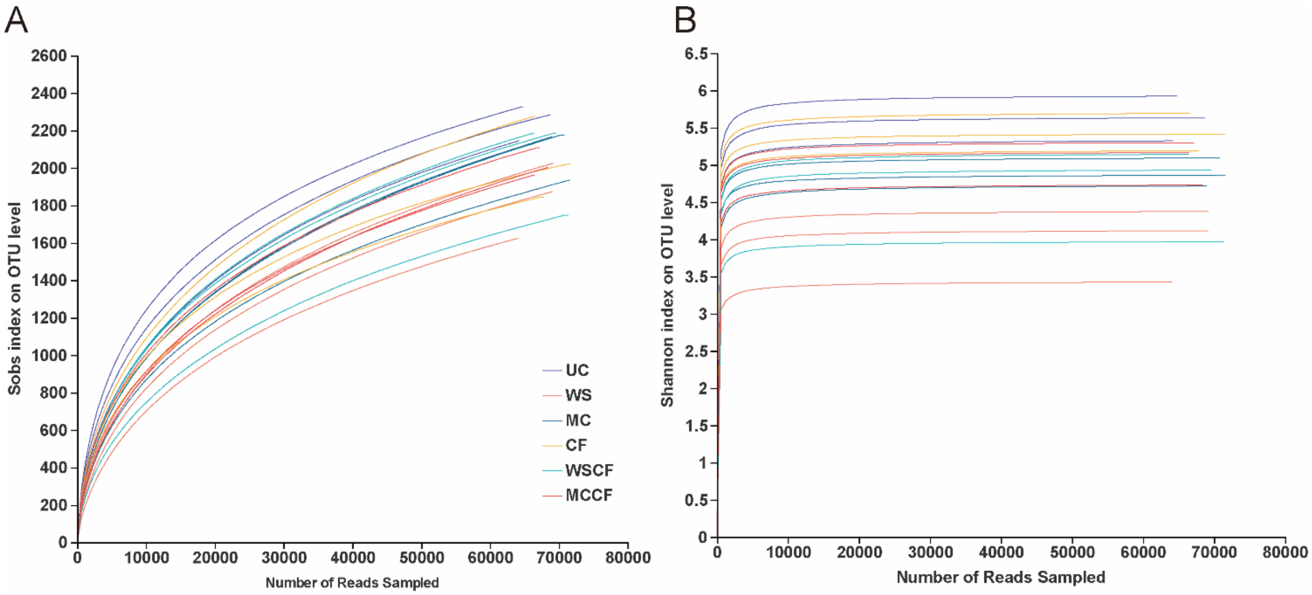

Fig S1. Rarefaction curve showing the relationship between fungal diversity and the number of random sampling sequences: (A) the number of operational taxonomic units (OTUs) observed in the experiment; and (B) the diversity predicted by the Shannon index

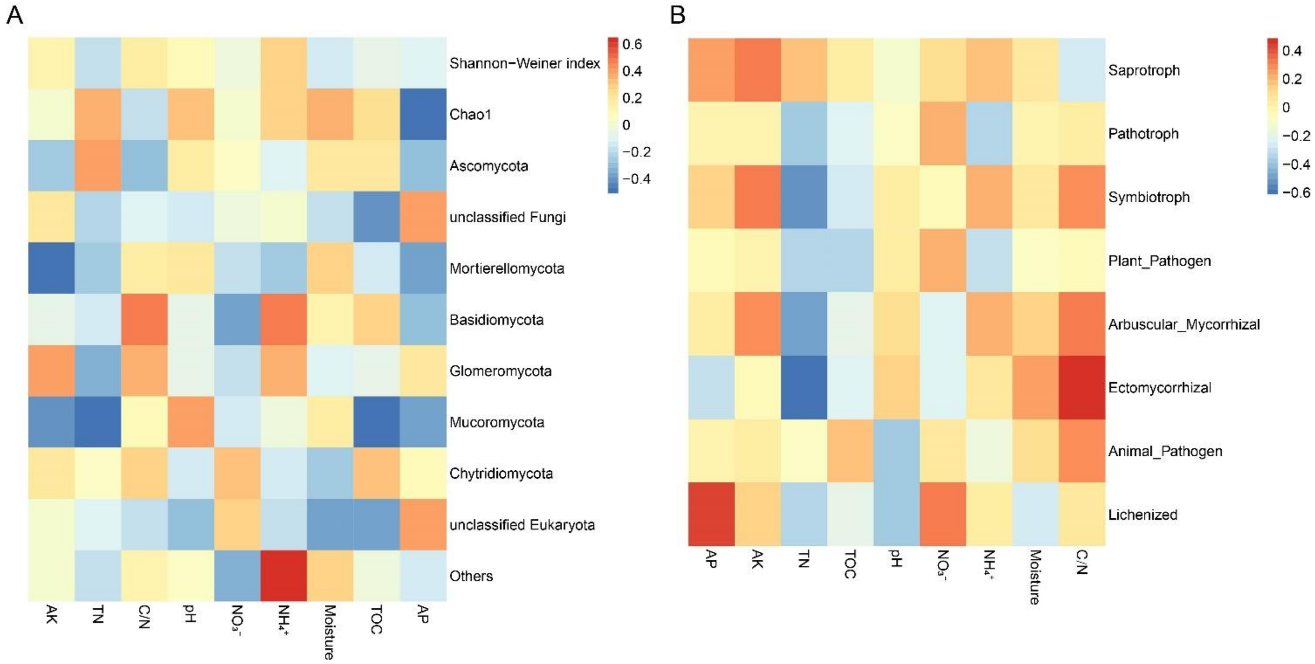

Fig S2. (A) Effects of soil physical and chemical properties on the soil fungal phylum; and (B) effects of soil physical and chemical properties on the functional groups of soil fungi. The x-axis and the y-axis are soil physical and chemical factors and fungal physic factors; correlation coefficients (R values) are shown in the figure in different colors; and the legend on the right is the color interval of different R values, with colors ranging from blue (negative correlation) to red (positive correlation). TOC, total organic carbon; TN, total nitrogen; AP, effective phosphorus; AK, effective potassium; NO_3^-, nitrous nitrogen; NH_4^+, ammonium nitrogen

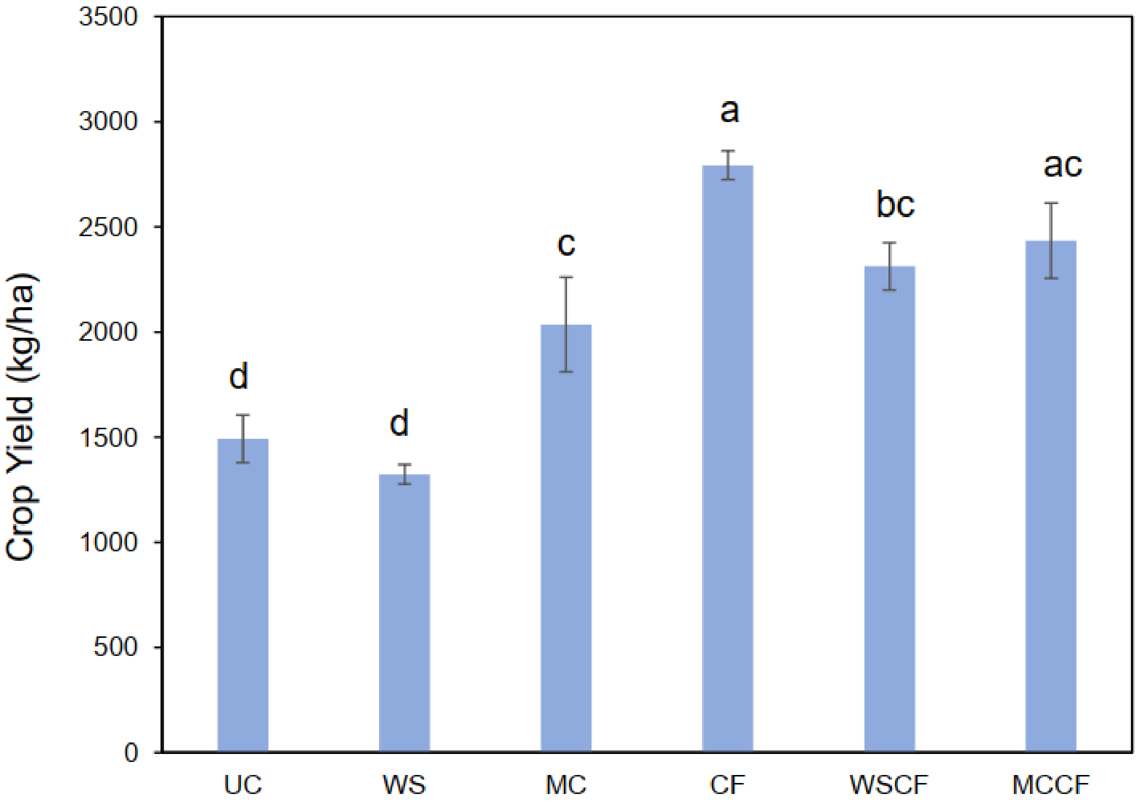

Fig S3. Effect of different fertilization on corn yield. UC, unfertilized control; WS, wheat straw; MC, manure compost; CF, mineral fertilizer (nitrogen, phosphorus, and potassium); WSCF, WS plus CF; MCCF, MC plus CF. The same letter indicates no significant difference, and different letters indicate a significant difference ($p < 0.05$)

Table S1 The amount of fertilizers used for different treatments (kg ha^{-1}). UC, unfertilized control; WS, wheat straw; MC, manure compost; CF, mineral fertilizer; WSCF, WS plus CF; MCCF, MC plus CF.

Treatments	nitrogen	P_2O_5	K_2O	wheat straw	cattle manure compost
UC	0	0	0	0	0
WS	0	0	0	6000	0
MC	0	0	0	0	10000
CF	180	90	90	0	0
WSCF	180	90	90	6000	0
MCCF	180	90	90	0	10000
